# Principles of Periocular Reconstruction following Excision of Cutaneous Malignancy

**DOI:** 10.1155/2012/438502

**Published:** 2012-12-17

**Authors:** Scott M. Hayano, Katherine M. Whipple, Bobby S. Korn, Don O. Kikkawa

**Affiliations:** Division of Ophthalmic Plastic and Reconstructive Surgery, UCSD Department of Ophthalmology, Shiley Eye Center, 9415 Campus Point Drive, La Jolla, CA 92093-0946, USA

## Abstract

Reconstruction of periocular defects following excision of cutaneous malignancy can present difficulties for oculofacial and reconstructive surgeons. The intricate anatomy of the eyelids and face requires precise restoration in order to avoid postoperative functional anesthetic concerns. Various reconstructive procedures based on common principles, location and size of the defect, can be applied to achieve restoration with the best possible functional and aesthetic outcomes.

## 1. Introduction

With advancing age, cutaneous malignancy around the eye becomes more prevalent. The most common skin cancers that present in the periocular region are basal cell carcinoma, squamous cell carcinoma, sebaceous cell carcinoma, and malignant melanoma [1]. These tumors are usually diagnosed by incisional biopsy. Following tumor removal in the periocular region, reconstruction of the defect requires understanding of the differences and uses of soft tissue flaps and skin grafts [2–9]. Flaps are usually preferred over grafts because homogeneity of skin color and texture more likely leads to better unification with surrounding tissue [10]. Many techniques have been described and the reader should familiarize themselves with the more commonly used procedures covered in this paper [2, 3, 6–9, 11]. Because the eyelid is a layered structure, appropriate layered reconstruction is essential, with the goal towards restoring periocular function and minimizing any postsurgical complications [4, 12]. Proper eyelid volume and shape should be strived for [4, 13]. 

## 2. Tumor Excision

Removal of periocular neoplasms requires clear surgical margins, which can be attained by Moh's micrographic surgery, frozen tissue examination, or permanent sections [14]. Frozen sections for certain types of tumors, such as melanoma and sebaceous cell carcinoma, can be difficult to interpret and may require formalin fixation to ensure complete tumor excision. Adherence of the tumor to the bony orbit necessitates additional techniques described below.

## 3. Orbital Bony Involvement

If the tumor is adherent to the periosteum, removal of the involved periosteal segment and burring of the underlying bone is typically all that is required. The patient should have ongoing tumor surveillance with imaging. If there is significant bony destruction by the tumor, osteotomy should be performed with removal of the involved segment [15]. Any sharp edges that result from removal should be rounded to prevent penetration of overlying soft tissue. Dural exposures should be covered, however, primary bone grafting should be avoided. 

## 4. Principles of Eyelid Reconstruction

Reconstruction of the eyelid following tumor excision is designed based upon the size and depth of the defect, the inclusion of the lateral canthus or medial canthus, and whether the lacrimal system is involved. Each physician will devise a plan for reconstruction based on one's familiarity, experience, and preference with different eyelid restoration techniques. The ultimate goal of every eyelid reconstruction is to create a stable eyelid margin, to ensure the eyelid has proper dimensions and tension when open and closed, to obtain eyelid symmetry with no rough or uneven internal surfaces, and to optimize aesthetics [16]. 

There are fundamental guidelines that should be followed with every reconstruction effort. First, the surgeon should assess the defect. Partial-thickness defects involve skin and orbicularis, whereas full-thickness defects extend from skin through conjunctiva. When using free grafts, a vascular source must be provided by either the anterior or posterior lamella. Free grafts replacing the anterior lamella must not be placed upon a free graft reconstructing the posterior lamella and vice versa because of the lack of a vascular supply. An orbicularis advancement flap can be interposed between two free grafts with success [17]. Defects involving the posterior lamella can be restored using grafts from the hard palate, nasal chondromucosa, upper tarsus (pedicle based or free), or ear cartilage [18]. The anterior lamella is best reconstructed by transferring neighboring tissue. Full-thickness skin grafts from the upper lid, inner upper arm, retroauricular, or supraclavicular may be used if there is insufficient adjacent tissue. Procedures that include lid sharing, such as a Cutler-Beard flap or Hughes tarsoconjunctival flap, should be avoided in children in the amblyogenic stage of development [19]. If the defect has been replaced with skin grafts, the new tissue must be properly anchored into place in order to avoid any postoperative eyelid malposition.

**Figure 1:**
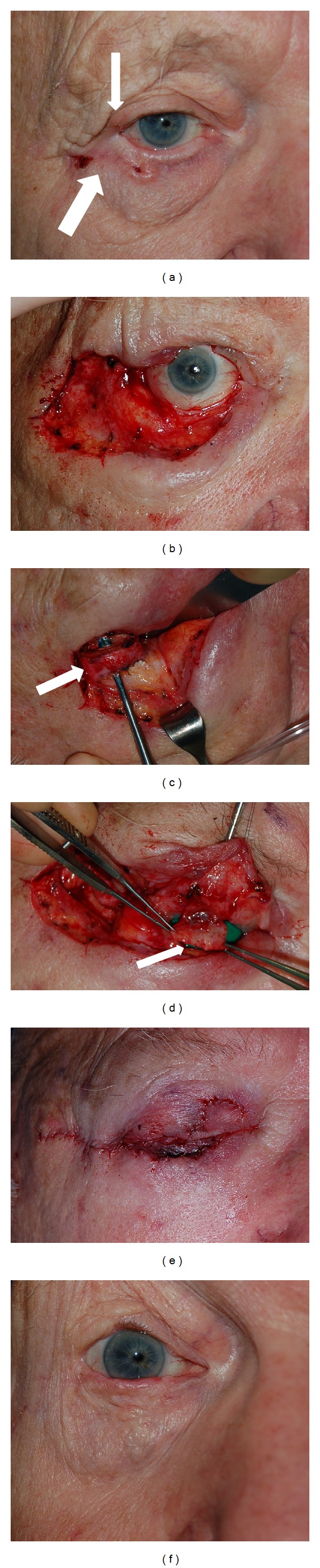
(a) Initial presentation of basal cell carcinoma in an 89-year-old patient involving the right lower eyelid (thick arrow) and right upper eyelid (thin arrow). (b) After Moh's resection, a large surgical wound measuring 5 cm by 2 cm involving 95% of the right lower eyelid and 60% of the right upper eyelid is present. (c) A periosteal flap was elevated from the lateral orbital rim and rotated medially to reconstruct the posterior lamella of the upper lid and provide an anchor for the lower lid. (d) A tarsoconjunctival flap was then harvested from the remaining superolateral tarsus of the right upper lid and rotated inferiorly to reconstruct the posterior lamella of the right lower eyelid (arrow). (e) Completed reconstruction with anterior lamellar full thickness skin graft from the left upper eyelid. (f) One year postoperative.

It is important to follow a systematic approach to eyelid reconstruction [20]. Eyelid margin defects can be closed using specific methods depending on how much of the horizontal lid length is removed. If the wound involves less than 20 percent of the upper or lower lid margin in younger individuals and up to 30 percent in older patients, it can be closed primarily. Extra length can be obtained by performing a lateral canthotomy and cantholysis. A Tenzel semicircular advancement flap can be used for reconstructing defects that include 25 to 50 percent of the upper or lower lid. For defects that involve over 50 percent of the lid margin of the lower eyelid, a Hughes tarsoconjunctival flap with a full-thickness skin graft or a Mustarde cheek rotational flap with a posterior lamellar graft can be used (Figure 1). The tarsoconjunctival flap is favored and can be opened in 2-3 weeks. Defects greater than 50 percent of the upper lid may use a Cutler-Beard pedicle or Leone flap [21].

## 5. Canthal Reconstruction

The medial and lateral canthi are sites where multiple aesthetic units overlap and present difficulties in attempting to preserve unique characteristics in that region [22]. Appropriate horizontal tension of the eyelid is important because it lowers the chances of eyelid malposition and exposure of the cornea [12]. Proper anchoring of the eyelid along the medial and lateral canthus is essential for proper function and aesthetics. Flaps using the periosteum can be used to rebuild canthal ligaments that structurally support the posterior lamella. Free skin grafts can be placed over periosteal flaps if used. One must take into account negative vectors, which necessitates that the periosteal flap must be located superiorly enough to the eye to avoid unwanted exposure [3, 7, 11, 12]. If part of the periosteum is removed during tumor excision, the canthi can be remodeled using a small titanium plate fixed with sutures instead. Small defects (less than 1 cm) can be allowed to heal via secondary intention.

## 6. Reconstruction of the Posterior Lamella

Reconstruction of the posterior lamella is best carried out using grafts from the tarsus [23]. Free grafts from the opposite eyelid's tarsus or a pedicle-based graft from the tarsus adjacent to the defect are preferred. The upper and lower tarsus is different in dimension, measuring 10–12 mm in the upper lid and 4-5 mm in the lower lid. Other alternatives are hard palate mucosa and ear or nasal cartilage. When a considerable amount of soft tissue is excised during tumor removal, dermis fat grafts may be used for restoring the deficient volume in order to ensure proper positioning of the eyelid [4, 24].

## 7. Reconstruction of the Anterior Lamella

**Figure 2:**
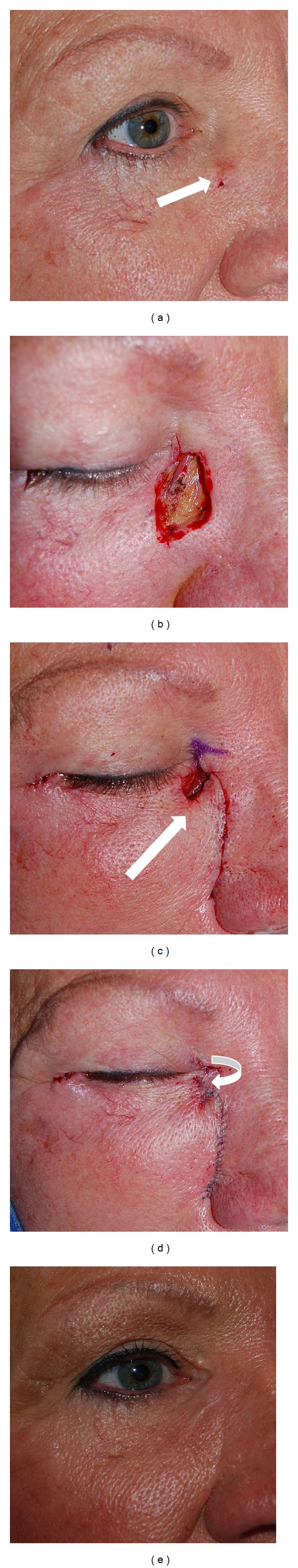
(a) Preoperative photo of a 62-year-old female with basal cell carcinoma of right medial canthus (arrow). (b) Local resection creating a 1.5 × 2.0 cm defect. (c) Adjacent tissue transfer flap created via an infraciliary incision and relaxing incision at the lateral aspect of the nose. The flap is then advanced superiorly and medially (arrow) to fill in approximately 85% of the wound. (d) A small rhomboid flap is elevated from the upper lid and rotated inferiorly to fill in the remaining wound (arrow). (e) One year postoperative.

**Figure 3:**
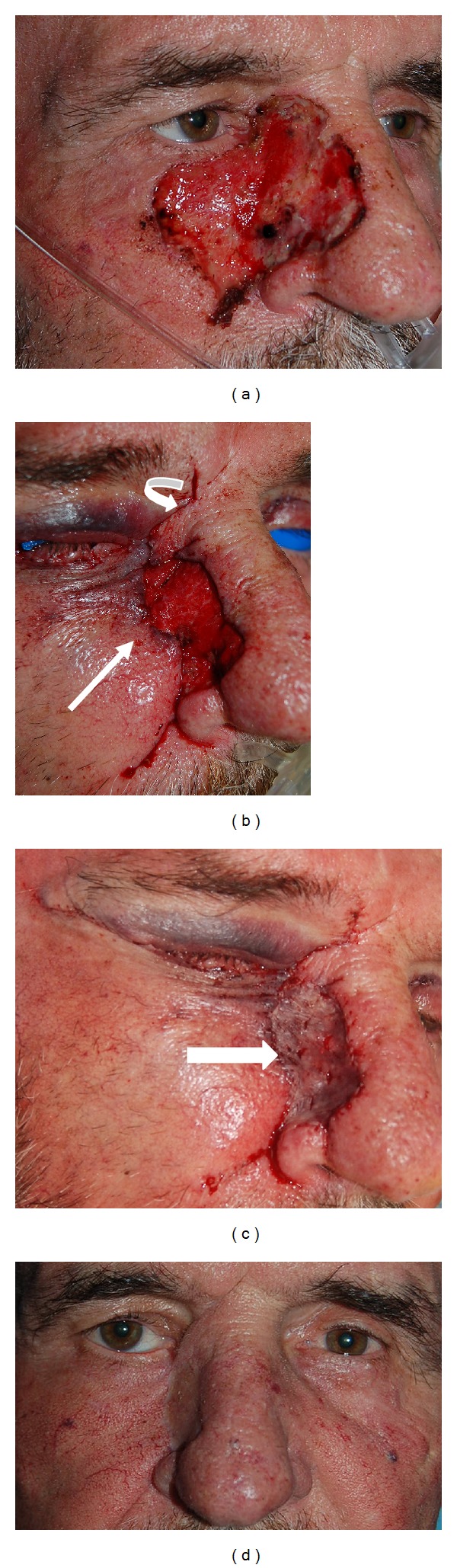
(a) a 65-year-old male status after Moh's resection for lentigo maligna melanoma in the right lower lid, cheek, and lateral nasal wall. The defect measured 5 cm by 4 cm. (b) It was decided to decrease the size of the defect using adjacent tissue flaps prior to considering use of a skin graft. Alternatively, a skin graft could be placed for the entire defect; however, it is our preference to use adjacent tissue whenever possible. An infraciliary incision with nasolabial extensions allows for the lateral midface and cheek to be rotated medially and superiorly to fill approximately 60% of the wound (straight arrow). In addition, this keeps the tension horizontal rather than vertical. A rhomboid flap is raised superiorly and rotated inferiorly to fill in the superior aspect of the wound (curved arrow). (c) A full thickness skin graft from the inner upper arm is used to fill in the remaining wound (arrow). (d) Eleven months postoperatively showing mild residual ectropion of right lower lid.

Neighboring tissue flaps are preferred because the tissue color and texture is most similar to the original tissue that was removed due to exposure to similar environmental conditions [2, 3, 7, 9, 14] (Figure 2). Alternative options include free skin grafts from the upper eyelid, retroauricular, supraclavicular, and inner arm (Figure 3). When there are insufficient full-thickness skin grafts obtainable, split-thickness skin grafts are acceptable. However, split-thickness grafts contract more postoperatively. Compared to the posterior lamella, the type of anterior lamella reconstruction influences more the final aesthetic outcome [3, 25]. Relaxing incisions for flaps are useful when made along adjoining aesthetic units. To avoid eyelid retraction and ectropion, vertical skin tension in the lower eyelid must be monitored [3, 7, 9, 11, 14].

## 8. Flap Design

**Figure 4:**
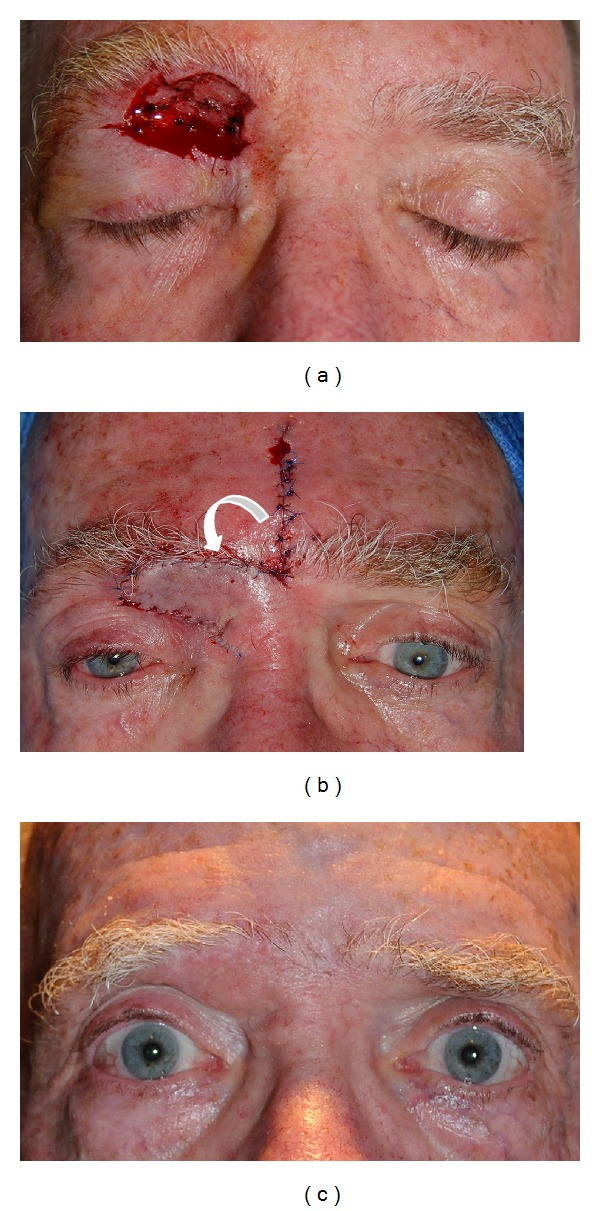
(a) A 67-year-old male status after Moh's excision of basal cell carcinoma of the superomedial right upper eyelid creating a 3 × 2.5 cm wound, which also included the preseptal and infrabrow region. (b) A glabellar flap is harvested centrally and rotated inferiorly and laterally to reconstruct the defect. Since the defect involved only the anterior lamella, no posterior lamella or muscle layer reconstruction was necessary. (c) 10 months postoperatively with an excellent result.

The face and eyelids have extensive vascular supply. Because of this, the design of rotational flaps based on a particular arterial supply is not necessary (Figure 4). The subdermal vascular plexus can provide adequate blood flow to support random flaps. Complications are more common in patients with a history of facial surgery, smoking, vascular disease, and radiation.

It is important to minimize horizontal tension, which is why incisions should be made in conjunction with relaxed skin tension lines [2, 5, 7, 9, 22]. This promotes proper wound healing and decreases the chances of tissue necrosis and eyelid malposition. Most of the time, the problem of tension is lessened due to the fact that many skin cancer patients are elderly with increased skin elasticity. Wound eversion is best achieved using mattress sutures and results in aesthetically pleasing closures compared to simple running sutures [26]. Deeper sutures in the superficial musculo-aponeurotic system (SMAS) layer can be used to minimize wound tension [2, 3, 11]. These sutures in the SMAS can also be placed in a “hang-back” fashion with deeper anchoring to the periosteum [3, 5–7, 24]. Midfacial defects require sutures that mimic the support of the orbitomalar ligament. 

## 9. Complications

Despite the surgeon's best effort, occasional complications may occur (see Table 1). Approximately 10 percent of cases may require additional surgery to correct lid malposition. This rate may be higher in patients with a history of radiation, smoking, and previous surgery. Secondary skin grafting may be required. Patients with large tumors or perineural invasion are at a particular risk for recurrence and the index of suspicion should remain high in these patients.

## 10. Conclusion

Reconstruction of periorbital defects following Moh's surgery requires special attention to both aspects of aesthetic appearance and eyelid function [2, 3, 7, 8, 12, 25]. Many different techniques and principles have been described, and the surgeon has some latitude to achieve the best result [2, 3, 6–8, 11, 25]. While it is important to follow a systematic reconstructive algorithm, it is our opinion that each patient is unique and a plan must be formulated for each individual following fundamental principles.

## Figures and Tables

**Table 1:** Complications of eyelid reconstruction.

Eyelid retraction	
Cicatricial ectropion or entropion	
Ptosis	
Lagophthalmos	
Dry eye	
Tumor recurrence	
Trichiasis	
Infection	
Graft failure	
Scarring	
Hyper or hypopigmentation	
